# Simulation Insights into the Assembly of Polyplexes for RNA Delivery

**DOI:** 10.1021/acs.biomac.5c01219

**Published:** 2025-11-19

**Authors:** Jonas Hans Lehnen, Jorge Moreno Herrero, Heinrich Haas, Friederike Schmid, Giovanni Settanni

**Affiliations:** † Department of Physics, 9182 Johannes-Gutenberg University Mainz, Staudingerweg 9, 55128 Mainz, Germany; ‡ 435117 BioNTech SE, An der Goldgrube 12, 55131 Mainz, Germany; § Faculty of Physics and Astronomy, Ruhr University Bochum, Universitätsstrasse 150, 44801 Bochum, Germany

## Abstract

RNA-based pharmaceuticals proved successful with the COVID-19 vaccines and are now undergoing clinical trials for a broad range of therapeutic indications. Lipid-based nanoparticles (LNPs) have been used so far as delivery systems, although alternatives are still needed to meet efficacy and safety requirements across a broader range of applications. Polyplexes, formed by the self-assembly of cationic polymers with the anionic nucleic acids, constitute a valuable substitute, especially if precise control of the number and shape of the encapsulated RNA chains is possible. Here, we use molecular dynamics simulations of a coarse-grained polyplex model to show that the most important factors controlling it are the charge ratio between polyelectrolytes and RNA and their concentration during assembly. Close to the isoelectric point, the polyplexes are large, whereas in large excess of cationic polymer, their size decreases, allowing one RNA copy per nanoparticle. Our results are consistent with recent experimental work on polyethylenimine polyplexes.

## Graphical abstract

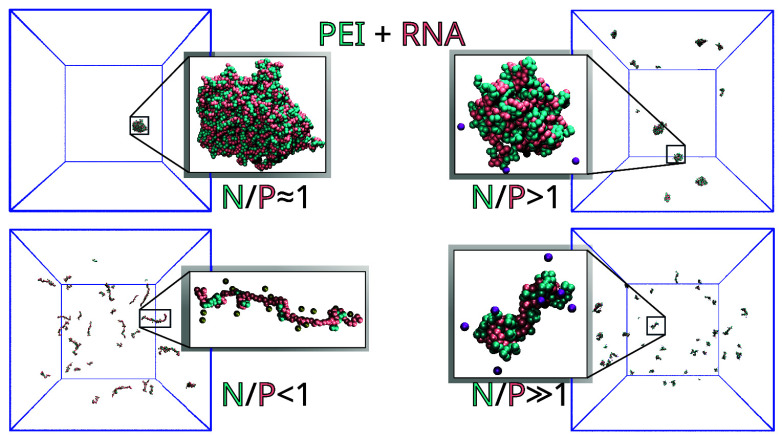

## Introduction

Pharmaceutical nanoparticle products based on RNA have been approved for several therapeutic and preventive applications. Lipid nanoparticles (LNPs) transporting silencing RNA (siRNA) are used to inhibit expression of particular genes as a therapy against several hereditary disorders.^1–4^ In the vaccines against COVID-19,^5,6^ LNPs deliver mRNA encoding for the spike protein of the SARS-CoV2 virus to antigen-presenting cells, stimulating an immune response against the virus. Several other products based on RNA are currently under investigation as immunotherapeutic agents for the treatment of tumors and other viral infections.^7^

Nanoparticles are used to protect the RNA from degradation and to deliver it to the target site. The current gold standard for delivery of siRNA as well as mRNA is LNPs, made up from a specific composition of four different lipids. These nanomaterials, however, only allow for a relatively small fraction of the nucleic acid to be effectively delivered,^8,9^ and some of their components may be related to the observed side effects.^10^ Although optimization strategies for more efficient LNP delivery are progressing,^7,11–16^ there is a need for improvements and an expansion of the cohort of available delivery systems with respect to efficacy, selectivity, immunogenicity, and safety, to help and address a larger variety of tissues and to lower the size of the required doses and, consequently, the incidence of side effects.

Polymers and, in particular, polycations have been investigated for more than two decades in connection with the advancements in nucleic acid therapies and have been proposed as a possible alternative to LNPs. Ionizable polycations like polyethylenimine (PEI) have been demonstrated to be successful for the delivery of nucleotide cargos, including DNA and different RNA formats.^17,18^ By the rapid mixing of polycations and nucleic acid chains, polyplexes are formed. In recent experiments, Moreno Herrero et al.^19^ systematically investigated the complexation of self-amplifying mRNA (saRNA) with PEI, where they elucidated the correlation between process conditions, particle size, and biological activity. They verified that a large ratio between PEI monomers and RNA nucleotides (N/P ratio) resulted in the formation of small nanoparticles, containing mostly one single RNA chain, and identified these nanoparticles as the actual basis for improved transfection efficiency in vivo.

Simulations of the interactions between nucleic acids and nanodelivery materials like lipid formulations^20–24^ or polycations^25^ have played an important role in the molecular understanding of these systems. Atomistic simulation approaches have been used to determine the mode of binding between PEI chains and double-stranded DNA or siRNA fragments.^26–29^ The complexity of this approach however has limited its use so far for studying the process of formation of polyplexes, which may include multiple long nucleic acid and PEI chains.^30^

Coarse-grained approaches help overcome these limitations by reducing the number of degrees of freedom and speeding up the dynamics. Different levels of coarse-graining are possible. Intermediate resolution approaches using MARTINI^31–33^ or MARTINI-like force fields^34^ have been employed. For example, Wei and Luijten^34^ built a MARTINI-like force field-based on atomistic simulations and studied the binding process of multiple PEI chains with a double-stranded nucleic acid fragment. Using the MARTINI approach, Tang and Mahajan^35^ investigated the aggregation kinetics and modes of binding of short PEI with RNA at varying N/P ratios as well as the effect of two different salt concentrations. In a subsequent investigation,^36^ they focused on the effects of PEI protonation on PEI-RNA polyplexes mimicking the acidification inside endosomes. More recently, Binder et al.^37^ used a titrable MARTINI model to investigate the complexation of PEI to siRNA. However, also with those approaches, the time and length scales of nanoparticle formation remained inaccessible.

In previous works involving one of us,^38,39^ a simple bead-and-spring model including electrostatic interactions was introduced and used to monitor the nanoparticle formation of a system composed of a single long polyanion, representing the nucleic acid, and a varying number of oligocations. It was demonstrated that an increase in the concentration of oligocations up to polyanion neutralization led to a collapse of the size of the polyanion. Further addition of polycations above the isoelectric point resulted in positively overcharged and partly swollen nanoparticles. A similar study^40^ investigated the role of several factors in the formation of polyplexes including polyanion and polycation chain length and stiffness, although the effect of relative chain abundance was not systematically considered. Using a relatively similar model, Chen et al.^41^ recently investigated the formation of clusters of polyanions and polycations in dilute solutions. They found that asymmetry in the concentration or length of the oppositely charged polyelectrolytes resulted in clusters with a net charge and that below a certain threshold in the asymmetry, the formation of large aggregates is observed. The study however did not cover the same parameter space as the experimental data on polyplex formation.^19^

Here, we investigate the behavior of a system containing multiple nucleic acid chains and systematically investigate the effects of different N/P ratios as well as polycation length and concentration on the aggregation and dissolution of polyplexes, and we show how the model provides a mechanistic understanding of the available experimental data.^19^

## Results and Discussion

### The Model

We adopt a coarse-grained model similar to the one presented in Zhou et al.^38^ to simulate RNA and linear polycations (named PEI below for simplicity) as charged flexible polyelectrolyte chains. Each RNA bead represents a nucleotide and is assigned a negative unit charge (−*e*) while a PEI bead represents two monomers and is assigned a positive unit charge (+*e*), resulting in a protonation ratio α = 50% which is close to the values reported previously.^42^ Monovalent ion-beads of opposite charge are added for every RNA and PEI bead in the system. Positively charged ions are referred to as counterions, while negatively charged ions are called co-ions. All beads in the simulation have the same mass *m* and diameter σ. The bond length between beads in PEI and RNA chains is also set to σ (Figure 1 top). For comparisons with experimental data, we assume σ = 0.6 nm as the average distance between phosphorus atoms of adjacent nucleotides in RNA chains. Excluded volume interactions between particles are modeled using the WCA potential,^43^ electrostatic interactions are modeled with the Coulomb potential, and chain connectivity is provided by harmonic bonds. The simulated systems are built by randomly inserting, with no overlap, *N*_RNA_ chains of length *l*_RNA_ and *N*_PEI_ chains of length *l*_PEI_ in the simulation box of volume *V*_box_ along with the corresponding co- and counterions. The molecular dynamics simulations are run at constant volume and temperature for 10^6^ reduced Lennard-Jones time units corresponding to 2·10^8^ time steps (see the Methods section for definition of Lennard-Jones time unit), which is sufficient to reach convergence in most of the examined conditions. Details of all the interaction terms, the simulation setup, and protocols are provided in the Methods section.

**Figure 1:**
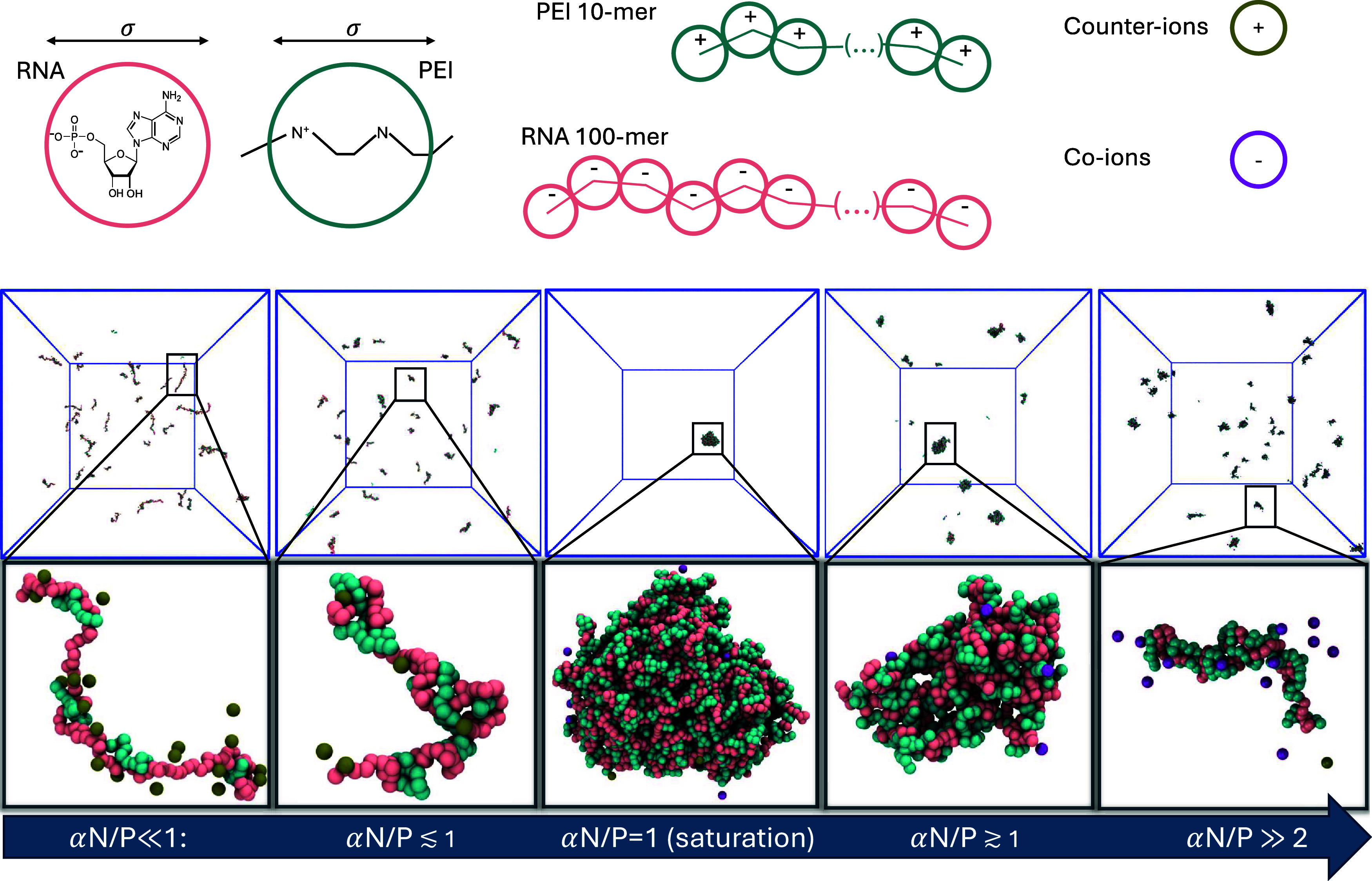
(Top) Schematic overview of the model reporting all of the species present in the simulations. (Bottom) Snapshots from the simulations at different values of the αN/P ratio, with close-ups on the typical nanoparticles.

An initial set of simulations was performed with the following parameters: *N*_RNA_ = 8, *l*_RNA_ = 100, *l*_PEI_ = 10, *V*_box_ = (200σ)^3^, and with *N*_PEI_ in the range [0–3000], which corresponds to an RNA nucleotide concentration of 0.7 mMol (ca. 0.23 mg/mL RNA) and a range of αN/P of [0.0:37.5]. Then, selected single parameters were varied as listed in Table 1 to determine their impact on the system.

**Table 1:** Parameters of the Simulations

varied parameter	range
*l*_PEI_	5, 8, 10, 12, 15, 20, 30
initial *n*_NPs_^*^ ^a^	≈0, 1
*V*_BOX_	(200–317.5 σ)^3^
*N*_RNA_	8, 16, 32^b^, 64^c^

a See below for the definition of *n*_NPs_^*^.

b Simulation reduced to 8·10^7^ time steps due to the large size of the system.

c Simulation reduced to 2·10^7^ time steps.

### Role of N/P Ratio in RNA Condensation/Aggregation

The simulations show that, in the absence of PEI, the RNA chains are stretched due to repulsive electrostatic interactions between monomers and only some counterions are bound to them, as reported by the hydrodynamic radius, shape anisotropy, and net average charge of the complexes and its contributions (Figures 1 and 2). The observed counterion condensation around RNA is in agreement with the theoretical expectations derived by Manning^44^ $\phi =1-\frac{\sigma }{l_{b}}$ , where ϕ is the fraction of polyelectrolyte charges neutralized by counterions and *l*_b_ is the Bjerrum length of the solution (*l*_b_ = 1.168σ ≈ 0.7 nm as in water, see the Methods section for details). When PEI is introduced into the system up to a αN/P ratio of 1 all of the PEI binds to the RNA chains. This results in a gradual collapse (condensation) of the stretched RNA and expulsion of counterions (Figures 1 and 2a–d) as the PEI reduces the net charge of the RNA-PEI complex and is able to form bridges between distant monomers of the RNA. This behavior is in agreement with that observed in similar studies for flexible polymer chains.^38,40,41^

**Figure 2:**
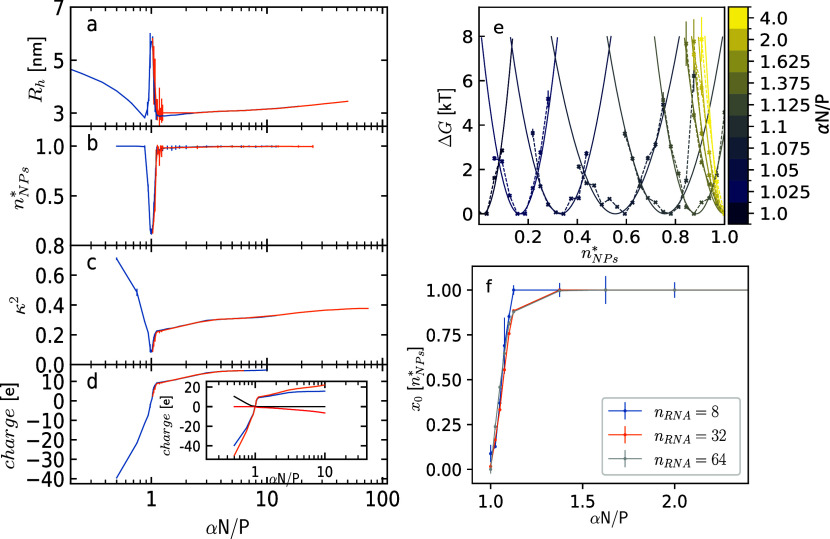
Average values of (a) the hydrodynamic radius *R*_h_, (b) the normalized number of NPs *n*_NPs_^*^, (c) the shape anisotropy κ^2^, and (d) the charge of the NPs as a function of the αN/P ratio. Data from simulations starting from *n*_NPs_^*^ = 1 and *n*_NPs_^*^ ≈ 0 are reported in orange and blue, respectively. Error bars indicate standard deviations. In the inset, the contributions to the total charge (blue) from the PEI and RNA (orange), co-ions (red), and counterions (black) are reported. (e) Free energy profiles as a function of *n*_NPs_^*^ for simulations including 32 RNA chains in the box data for the simulations with 8 and 64 RNA chains are reported in Figure S1. The dashed lines show simulation data, while the solid lines represent the parabolic fits. The color scale from blue to yellow indicates increasing αN/P ratios starting from αN/P = 1 with blue (see the color bar). (f) Position of the minimum of the parabolic fits in (e) as a function of αN/P ratio for simulations including 8, 32, and 64 RNA chains (but the same RNA concentration) in blue, orange, and light gray, respectively.

As the αN/P ratio of the system approaches 1, that is, the isoelectric point, the formation of large, almost spherical NPs (aggregation) containing multiple RNA chains is observed (Figures 1 and 2a–d). The maximal aggregation is reached at the isoelectric point, where all PEI and RNA form one large NP in the simulations, and no ions are bound to the NP (Figures 1 and 2a–d). This behavior has been already observed^40,41,45,46^ and associated with coacervation. The phenomenon is linked to the overall decrease of electrostatic repulsion between neutral clusters of polyelectrolytes and release of counter- and co-ions.

Increasing the amount of PEI further leads to the gradual dissociation of the large NP into several smaller ones driven by an excess of positive charge on the NP due to attached PEI, which is only partly compensated by co-ions (Figure 2d, inset). At αN/P ratios larger than 3 multi-RNA NPs become very rare, and at αN/P ratios larger than 15, only single-RNA NPs are observed in the simulations, as shown in Figure 2b, which reports the number of NPs normalized by the number of RNA chains *n*_NPs_^*^. This is in agreement with a similar observation by Gallops et al.^40^ for flexible chains at N/P = 4. In this regime, the excess PEI on the NPs increases only slowly with the αN/P ratio and its positive charge is partly compensated by the binding of co-ions, which keeps the overall charge of the NPs almost constant (Figure 2d inset). In addition, possibly due to the positive overcharge, NPs show a less spherical and more elongated shape in this regime (Figures 1 and 2c), similar to that observed in Zhou et al.^38^

The different phases of the system observed as a function of the relative concentration of cationic and anionic polymers could also be observed when starting the simulations from the aggregated state, demonstrating that the length of the simulations is sufficient for convergence (Figure 2a–d). The behavior observed in the present simulations is consistent with experimental observations^19^ as it will be discussed in the next sections. Some similarities to our observations have also been reported in the simulations of both the formation and the acidification process of PEI-DNA nanoparticles using the MARTINI force field.^35,36^ There, the largest tendency to aggregation was found around αN/P ≈1, although no system-wide aggregation was observed, either due to simulations being performed not close enough to the isoelectric point, or to the shorter nucleic acid chains considered, or to differences in the model (number of counterions, treatment of the long-range Coulomb interactions, etc.). Mahajan and Tang^35^ also reported a linear increase of PEI per nucleic acid chains in the aggregates at αN/P ratios up to 1 and a slower increase for larger αN/P ratios, similarly to that observed in the present work (Figure 2a–d). The effective Debye length in the simulations, obtained by measuring the decay length of the electrostatic potential around the polyplexes (Figure S8 in Supporting Information) shows an overall monotonic decrease which depends on the N/P ratio. Only at the isoelectric point, a small peak of the Debye length is observed, related to the depletion of charged polyelectrolyte chains from the internanoparticle space due to aggregation.

The number of nanoparticles per RNA molecule gives a convenient state variable, *n*_NPs_^*^ (inversely proportional to the average size of NPs). The simulations show fast dynamics and a large number of transitions between the states of the system, as identified by *n*_NPs_^*^. This allows us to derive a projection of the free energy of the system along *n*_NPs_^*^ at varying αN/P ratios (Figures 2e and S1 in the Supporting Information). As discussed before, at αN/P = 1 (isoelectric point), we find one or very few large NPs containing all PEI and RNA in the simulation. Accordingly, the free energy profiles for αN/P ratios close to 1 have their free energy minimum at *n*_NPs_^*^ ≈ 0 in all cases. With increasing αN/P ratio, large NPs tend to break up and, therefore, the minimum of the free energy shifts to higher *n*_NPs_^*^ until, for αN/P ratios larger than a certain threshold, single-RNA NPs (*n*_NPs_^*^ = 1) become the most observed state of the system. The shape of the free energy curves suggests fitting them, as a first approximation, with a quadratic function centered at the minimum. The position of the minimum of the parabola as a function of αN/P ratio increases linearly for small αN/P ratios with a slope that is independent of the number of RNA chains in the simulations (Figures 2f and S1 in the Supporting Information). For large αN/P ratios, the position of the minimum converges to *n*_NPs_^*^ = 1 in all cases. We do not observe any significant finite size effect.

### Role of PEI Length

The overall behavior of the system does not show to change dramatically as a function of the length *l*_PEI_ of the PEI chains (Figure S2 in the Supporting Information), with the exception of very short chains (*l*_PEI_ < 8) where full aggregation is not observed. We observe RNA condensation below the isoelectric point and dissociation for large αN/P ratios, and for *l*_PEI_ ≥ 8, a size peak at the isoelectric point (Figure S2 in Supporting Information). According to the free energy projections over the *n*_NPs_^*^ variable (Figures S3 and S4 in Supporting Information), for *l*_PEI_ = 8, the transition from aggregated to dissociated state occurs over a larger range of values of the αN/P ratio than for longer PEI chains and with larger size fluctuations resulting in almost flat free energy profiles for αN/P ratios close to the transition midpoint. For longer PEI chains, we observe narrower distributions of *n*_NPs_^*^ as shown in Figure S4, and the transition midpoint is shifted to lower αN/P ratios. With an increase in *l*_PEI_ above 15, a slowdown of the aggregation and dissociation dynamics of the NPs for αN/P ratios close to the transition midpoint results in scattered distributions of *n*_NPs_^*^ (Figure S5 in the Supporting Information). The slowdown is possibly related to the larger electrostatic binding interactions of longer PEI chains with RNA leading to a slower release after aggregation. The PEI-length-dependent shift of the aggregation midpoint toward lower αN/P ratios observed in the present simulations (*l*_PEI_ = 8 to *l*_PEI_ = 10 and larger) resembles the PEI-length-dependent shift of the amount of free saRNA in supernatant, corresponding to dissociated RNA chains, observed in centrifugation and UV diffusion experiments.^19^ The absence of aggregation at *l*_PEI_ = 5 can be ascribed to the fact that entropy loss upon binding of short PEI chains to RNA is not compensated by a sufficient enthalpy reduction, as reported by the negative ζ-potential of NPs (see below), indicating that the bound PEI chains are not sufficient to neutralize RNA.

In the simulations with *l*_PEI_ ≥ 8, we observe a positive overcharge of the NPs for αN/P ratios larger than 1 (Figure 2d), which results in a positive ζ-potential (Figure S2b). This is due to the aggregation to the NPs of an excess of 1 to 2 chains of PEI above the number needed to neutralize the charge of RNA (Figures S6 and S7 in Supporting Information). The resulting overcharge increases with αN/P ratio and with PEI length (Figure S2 in Supporting Information).

### Role of RNA and PEI Concentration

In the simulations, slightly higher RNA and PEI concentrations are used than in the experiments.^19^ Therefore, it is important to assess how those concentrations influence the behavior of the system. Simulations at several different RNA concentrations were performed for a range of αN/P ratios. The data (Figure 3a,c) show that for αN/P ratios below 1, neither the RNA concentration nor the PEI concentration influences NP size or shape anisotropy significantly and the system is fully characterized by the αN/P ratio. Under these conditions, all cationic polymers are bound to RNA chains, thereby fully contributing to the NP charge and aggregation behavior of the system, which is then solely characterized by αN/P.

**Figure 3:**
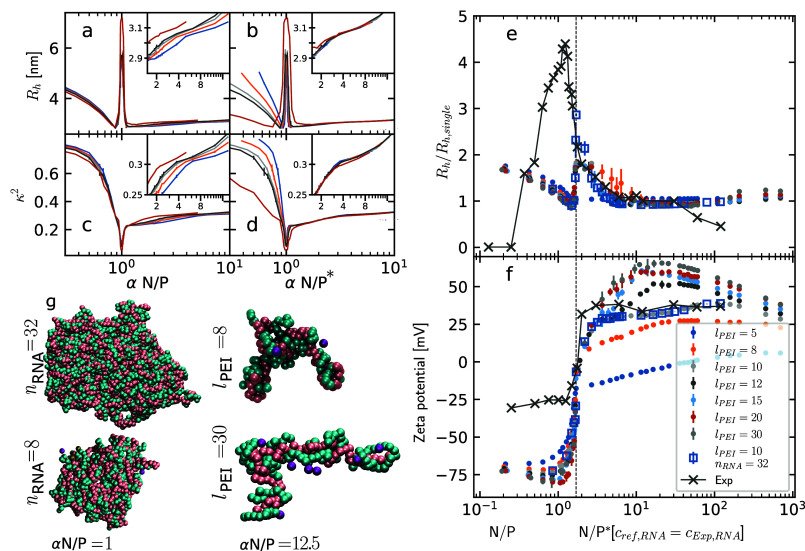
Hydrodynamic radius (a, b) and relative shape anisotropy (c, d) of the NPs for different RNA concentrations vs the αN/P ratios (a, c) and the excess αN/P* ratio (b, d). The colors indicate simulations at different *c*_RNA_. Simulations include 8 RNA chains with the exception of the dark-red line including 16 chains. (e) Size of the NPs as a function of N/P or N/P* ratio relative to the size of single-RNA NPs (at N/P* = 12). The experimentally measured Z-average from DLS experiments^19^­(dark gray, X markers) and hydrodynamic radius (*R*_h_) from the present simulations with 8 RNA chains (dots) and 32 RNA chains/*l*_PEI_ = 10 (squares) are reported. The N/P* ratio of experiments has been computed by considering the experimentally observed isoelectric point at N/P_iso_ = 1.7, from the ζ-potential. Stretched RNA chains, as observed in the simulations at N/P ≪ 1.7, do not diffract light efficiently for DLS measurements. (f) ζ-potential as a function of N/P and N/P* ratio from the experimental measurements^19^ and from the simulations. (g) On the left, snapshots of the fully aggregated NPs at αN/P = 1 for systems with 8 and 32 RNA molecules. On the right, snapshots of single-RNA NPs from simulations with different PEI lengths at a high N/P ratio and *c*_PEI_ comparable to experiments^19^ (N/P* > 200 in (e, f)).

In excess of cationic polymers (αN/P > 1), a part of the polymers aggregates onto the NPs making the NPs positively charged, while the rest remains free in solution. Under these conditions, both size and shape anisotropies show an apparent dependence on RNA concentrations and not only on the αN/P ratio (Figure 3a,c). The behavior of the system is dependent on the overall concentration *c*_PEI_^*^ of polycations exceeding those necessary to neutralize the RNA. This excess of polycations contributes both to the overcharge of the NPs, to the binding of co-ions and to the electrostatic screening around the NPs (Figure S8 in the Supporting Information)

1 $$c_{PEI}^{*}=\frac{N_{PEI}l_{PEI}-N_{RNA}l_{RNA}}{V_{box}}=c_{PEI}-c_{RNA}$$

To understand how this quantity is affected by the concentration of RNA­(*c*_RNA_), we compare it to a reference RNA concentration *c*_ref,RNA_. Then, by shifting the resulting number to match the isoelectric point, we can define the excess N/P ratio

2 αN/P∗=cPEI∗/cref,RNA+1=(cPEI−cRNA)cref,RNA+1=(αN/P−1)cRNAcref,RNA+1

This expression reports the excess polycation content (i.e., the polycation content above the one necessary to neutralize RNA) as a function of the RNA concentration and the overall αN/P ratio of the formulation. Then, we set 1/α = N/P_iso_, where N/P_iso_ is the N/P ratio where the sum of the electrostatic charges of the cationic and anionic polymers is exactly zero (this equals 1/α exactly in the simulations, while, in the experiments, it corresponds to the N/P ratio where the ζ-potential changes sign)

3 $$N/P^{*}=\left(N/P-N/P_{\mathrm{iso}}\right)\frac{c_{\mathrm{RNA}}}{c_{\mathrm{ref},\mathrm{RNA}}}+N/P_{\mathrm{iso}}$$

Now, the data plotted over the excess N/P* ratio (Figure 3bd) collapse on a single curve, indicating that the behavior of the system above the isoelectric point (αN/P > 1) is determined solely by N/P* and not by the RNA concentration. Thus, eq 3 allows for scaling the results of data obtained at different RNA concentrations and protonation levels and comparing them with one another.

### Comparison with Experimental Data

Considering that the concentration dependence of the simulation data can be factored out by using either the αN/P ratio or N/P*, depending on being either below or above the isoelectric point, respectively, we can compare our simulation results to the experiments. From the functional point of view, the region with N/P ≫ 1 is the most interesting, as in that region, the NPs were found to show the largest transfection efficiency.^19^ Since PEI is ionizable, its charge per monomer depends on the environmental conditions (pH, vicinity to negative charges, etc.). The experimental isoelectric point as returned by the ζ-potential (Figure 3f) occurs at an N/P ratio of 1.7, which then represents the αN/P_iso_ of the experimental data. The comparison between simulation and experimental data shows good qualitative agreement above the isoelectric point (Figure 3). At the isoelectric point, in our simulations, a single large NP is formed containing all PEI and RNA chains in the simulation box; therefore, the size or *R*_h_ of the NP is limited by the amount of RNA chains in the system. Increasing the number of RNA chains in our simulation also increases the height of this peak (Figure 3e,g). In the experiments, the available RNA chains are only few orders of magnitude less than Avogadro’s number; therefore, the peak in *R*_h_ at the isoelectric point is larger than in the simulation. Below the isoelectric point, an apparent discrepancy is observed between DLS measurements, which indicate a decrease in size, and the simulations, which indicate an increase in size due to the electrostatically driven stretching of the RNA chains. The discrepancy however is related to the fact that extended RNA chains do not scatter light efficiently in DSL experiments. Indeed, SAXS data reported in ref 19 show that the radius of gyration of RNA particles in the absence of PEI (N/P = 0) is significantly larger than those of monomeric RNA particles at N/P = 12, as we also observe in the simulations (Figure S2 in the Supporting Information).

The ζ-potential measures the electrostatic potential at the slipping surface of a particle. It is comparable to the potential generated by the charges from the NP itself and the ions that are strongly bound to its surface. In Figure 3f, the electrostatic potential measured on the simulated NPs (including attached ions at a distance smaller than 3.5σ from any PEI or RNA bead of the NP) is compared to the experimentally measured ζ-potential. In both experiments and simulations, with the exception of *l*_PEI_ = 5 discussed later, as the N/P ratio increases, the ζ-potential increases from a negative to a positive value and crosses the point of zero potential at the isoelectric point, as shown in Figure 3f. Above the isoelectric point, the potential reaches a nearly constant positive value. In the simulations, the height of the positive plateau is dependent on PEI length, as it is directly related to the overcharge discussed above. The PEI-length-dependent overcharge is also responsible for the change in size and shape of NPs (Figure 3g). The data from the simulations with PEI lengths above 12 show a good agreement with the experimental data in terms of the relative difference between the positive and negative plateaus of the ζ-potential, further validating the goodness of the model. In the case of *l*_PEI_ = 5, the ζ-potential remains negative even above the isoelectric point, indicating that the RNA chains are not being fully neutralized by bound PEI chains. As mentioned above, this is an effect of the entropy loss upon binding not being compensated for by an enthalpy reduction due to the short chain. This phenomenon is also observed in the experiments,^19^ where very short PEI chains require a large N/P ratio to induce aggregation.

## Conclusions

We have modeled the complexation process between cationic polymers and RNA by means of a very simple coarse-grained mechanistic model where RNA and cationic polymer chains are represented by anionic and cationic strings of impenetrable beads surrounded by neutralizing co- and counterions, and interacting with an effective electrostatic potential. No model parameters were fitted to the experimental data. Only the imposed equivalence between anionic and cationic bead sizes provides a rather weak constraint on the chemical nature of the cationic polymers. Thus, while we have compared the model results to experiments involving linear PEI,^19^ the model is not restricted to PEI, and it may be applicable to a range of linear cationic polymers. Indeed, the same experiments in ref 19 show that similar N/P ratio-dependent results can be reproduced with a large variety of different cationic polymers. We believe that the mechanisms for complexation follow general principles of self-assembly between oppositely charged polyelectrolytes as described in the literature.^46^ Our main results can be summarized as follow: in excess RNA (small N/P ratio), complexes with PEI form, but the small amount of PEI does not compensate the repulsive interactions between the negative charges of single RNA chains which remain separated and stretched; when the PEI concentration reaches values which allow to neutralize the RNA charges (isoelectric point) the electrostatic repulsion between RNA chains is abolished and large multi-RNA NPs can form; this aggregation occurs only if the cationic polymer chains are longer than a certain minimum, determined by a balance between entropy loss and enthalpy gain upon binding to RNA. Finally, in excess PEI, a positive overcharging of the PEI-RNA complexes leads to a reduction of the size of the NPs and the amount of RNA chains contained in each of them, up to the point where only one single RNA chain is contained in each NP. These results are consistent with available experimental data from Moreno Herrero et al.^19^

The model has been specifically built considering linear PEI polymers because this is the format which, being available in GMP grade, is mostly used for transfection and pharmaceutical applications in clinical trials (source https://clinicaltrials.gov/ accessed 9 Oct 2025). Linear PEI is also considered less toxic than branched PEI,^47^ which is an important advantage for application in pharmaceutical products. Branched constructs have also been used as delivery material,^48^ although experimental characterizations of polyplex assembly for these polymers with mRNA or saRNA are less abundant than in the linear case.

Given the simplicity of the model, the RNA-cationic polymer interactions beyond steric hindrance and electrostatics are not taken into account. The specific chemical structure of the cationic polymers may help modulating these interactions for example by providing specific binding for the structural motifs of the nucleic acids or variations to the hydrophilicity/hydrophobicity profile and/or the stiffness of the polymers with an influence on nucleobase compaction as shown in atomistic or MARTINI simulations,^29,37,49^ which, however, are too computationally expensive to simulate full polyplex assembly in the presence of multiple RNA chains as done in the present work. Also, the ionizable nature of the cationic polymers in the present simulation has been approximated by fixing the value of the fraction α of charged monomers. Constant pH approaches may help modulate further the interactions between the polymers and the RNA by allowing changes in the protonation state of monomers depending on the surrounding environment.^37,50^ It is worth noting, however, that these approaches may considerably add computational complexity.

The model presented here provides a mechanistic framework to elucidate the assembly of cationic-polymer-based nucleic acid delivery vehicles. It also offers, in perspective, the possibility for a detailed investigation of the kinetics of the assembly process, which may help improve polyplex preparation techniques aimed to decreasing cytotoxicity, increasing RNA encapsulation, and reaching larger transfection efficiencies with less side effects. Thanks to its simplicity and general appeal, the model opens the way to study polyplex behavior upon changes in environmental conditions such as pH (due to storage, injection into the bloodstream, or endosomal uptake). It also lends itself to an extension to branched polymers, provided that additional parameters describing the branching are included.

## Methods

### Molecular Dynamics Simulations

Short-range excluded volume interactions are modeled with the repulsive part of the Lennard-Jones potential (Weeks–Chandler–Anderson potential or WCA^43^)­

4 $$V_{\mathrm{WCA}}=\{\begin{array}{l} 4\varepsilon [{\left(\frac{\sigma }{r}\right)}^{12}-{\left(\frac{\sigma }{r}\right)}^{6}],,\mathrm{if}\;r<2^{1/6}\sigma \\ 0,,\mathrm{if}\;r\geq 2^{1/6}\sigma \end{array}$$

where *r* is the distance between two beads, σ is the distance where the original Lennard-Jones potential equals zero, and ε characterizes the energy scale. One Lennard-Jones time unit corresponds to the time $\sqrt{\varepsilon /m\sigma^{2}}$ , where *m* is the mass of the particles. A time step d*t* = 0.005 Lennard-Jones time unit has been used for all the simulations. The beads in the RNA and PEI chains are connected by a harmonic spring of the form

5 $$U{\left(r\right)}_{\mathrm{bond}}=\frac{1}{2}k_{\mathrm{bond}}{\left(r-\sigma \right)}^{2}$$

where *r* and σ are equivalent to the ones given above. The interaction strength is set to *k*_bond_ = 5000εσ^–2^. For these choices, the bond length fluctuates within 10% of the equilibrium bond length, and chain crossing is prohibited. The charges on the beads interact with each other through the Coulomb potential, which can be written as

6 $$U_{\mathrm{Coulomb}}\left(r\right)=\frac{e^{2}}{4\pi \epsilon_{r}\epsilon_{0}r}=k_{B}T\frac{l_{B}}{r}$$

where ϵ*_r_* represents the dielectric constant of the medium (water), ϵ_0_ is the vacuum permittivity, *k*_B_ is the Boltzmann constant, and T is the temperature. We treat both RNA and PEI as strong polyelectrolytes and set a temperature of *k*_b_ *T* = 1ε as done in Zhou et al.^38^ In this work, we use a Bjerrum length *l*_b_ = 1.168σ, which allows for fast relaxation dynamics and a direct comparison with experiments where the Bjerrum length of water is *l*_b_ ≈ 0.7 nm at room temperature and pressure. This ensures that the model temperature corresponds to experimental room temperature. We use a leapfrog algorithm together with the v-rescale thermostat^51^ to simulate a canonical NVT ensemble. Short-range electrostatic interactions are cut off at 10σ. Long-range electrostatic interactions are treated using smooth PME^52^ with an initial Fourier spacing of 2.0σ. Simulations are carried out using the program GROMACS 2022.^53^ In GROMACS, the Coulomb potential is implemented as

7 $$V_{c}\left(r_{\mathrm{ij}}\right)=f\frac{q_{i}q_{j}}{\epsilon_{r}r_{\mathrm{ij}}}$$

with *q*_*i*_ representing the charges of the particles, *r*_*ij*_ the distance, and *f* = 1/(4πϵ_0_) = 138.935458 mol^–1^ nm e^–2^. To perform the simulations with our reduced units, we set ϵ_*r*_ = 138.935458/(*l*_B_/σ). To work at our desired temperature *T* = ε/*k*_b_, we set the reference temperature in GROMACS input file to 120.27236 K. To minimize the total error of the calculated Coulomb forces, we use the “gmx pme_error”^54^ function to optimize the PME parameters before starting the simulation. This results in a typical grid spacing between 2σ and 3.25σ after GROMACS optimization during the run. PEI and RNA chains and the neutralizing ions were placed randomly in two separate simulation boxes, and overlaps between beads were relaxed with 100 time steps of molecular dynamics simulation using a soft core repulsive potential. The resulting concentration of positive counterions ranges between 0.35 and 0.7 mM, and the one for negative co-ions between 0 and 26.5 mM. Afterward, the energy was minimized and equilibrated for 2·10^6^ time steps. Subsequently, both systems were mixed and equilibrated again as described above. The simulations were then run for 2·10^8^ time steps with a integration constant of 0.005*t* (reduced Lennard-Jones time units). Four replicate runs were performed for most of the simulated systems, with the exception of the simulations with *n*_RNA_ = 32, where 3 replicates were performed, and *n*_RNA_ = 64, where 2 replicates were run. Replicate runs differ in the initial positions and velocities of all polymers and ions. Only in the simulations from the aggregated state is the initial conformation of the aggregated nanoparticle the same in all replicates, but the positions of added ions and PEI chains were assigned differently in each replicate.

A further set of simulations was started from the state where all RNA chains in the box are aggregated in a single NP including the PEI. This state corresponds to the final state of the simulations at αN/P = 1 from the other sets of simulations described above. Subsequently, PEI and neutralizing co-ions were added to the system to reach the expected αN/P ratio, minimized, and equilibrated before starting the production run, as described above.

Although the simulations are performed without explicit solvent or Langevin thermostat, the diffusion of the NPs in the simulations has been quantified (Figure 4) and shows that for sufficiently large time scales (τ > 10^4^ *t*) they undergo diffusive dynamics, that is, MSD­(τ) ∝ τ, due to collisions with the free ions and PEI chains in the system. We note that introducing a Langevin thermostat artificially alters the diffusion of large particle aggregates, which, due to the uncorrelated random forces acting on both surface and inner particles, will virtually stop diffusing. For this reason, we avoided using a Langevin thermostat. Explicit hydrodynamic interactions using dissipative particle dynamics (DPD) were not included, as that would have slowed down the calculations significantly.

**Figure 4:**
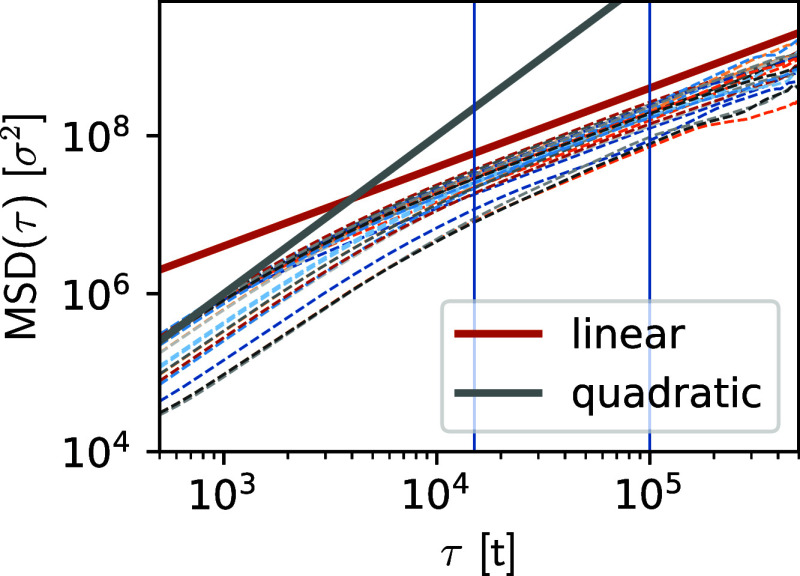
Mean square displacement of the RNA beads for different αN/P ratios and RNA concentrations (dashed lines of different colors). Solid gray and brown lines represent references to the quadratic and linear regimes, respectively. The blue vertical lines indicate the range of τ where MSD is linear in τ, indicating Brownian motion for NPs.

### N/P Ratio and Derivatives

The N/P ratio is the ratio between the number of nitrogen atoms from the amine groups of PEI and the number of phosphate atoms from RNA. For a system of charged polyelectrolytes, the more important quantity is the charge ratio between the species. In this paper, we assume that every polycationic bead (representing two PEI monomers) is charged, unless specified otherwise. With α = 50% as the protonation ratio of PEI, this gives

8 $$\alpha N/P=\frac{N_{\mathrm{PEI}}l_{\mathrm{PEI}}}{N_{\mathrm{RNA}}l_{\mathrm{RNA}}}$$

Unless specified otherwise, the protonation is constant at α = 50% and every change in αN/P is a result of a change in PEI or RNA concentration in the simulation.

Together with either the RNA or PEI concentration, the αN/P ratio is sufficient to characterize the molecular content of the system. Alternatively, the concentration of both repeat units (monomers) of the polyelectrolytes could be used.

9 $$c_{\mathrm{PEI}}=\frac{N_{\mathrm{PEI}}l_{\mathrm{PEI}}}{V_{\mathrm{box}}}$$

10 $$c_{\mathrm{RNA}}=\frac{N_{\mathrm{RNA}}l_{\mathrm{RNA}}}{V_{\mathrm{box}}}$$

with *V*_box_ being the volume of the solution or the size of the simulation box.

### Definition of a Nanoparticle

PEI and RNA self-assemble into NPs in the simulations. An NP is defined as the set of all PEI and RNA chains closer than a cutoff distance of 3.5σ to another member of the NP. Thus, the NP assignment process is iterative. Several properties of the NPs have been monitored along with the simulations

### Gyration Tensor

The gyration tensor of an NP is defined as

11 $$S=\frac{1}{N}\sum_{i=1}^{N}\left(r_{i}-r_{\mathrm{com}}\right)⊗\left(r_{i}-r_{c\mathrm{om}}\right)l$$

where *r*_com_ is the center of mass of the NP, and the sum runs over all the beads in an NP. The gyration tensor is a symmetric 3 × 3 matrix and can be diagonalized by a proper rotation. We denote the eigenvalues as λ_1_^2^, λ_2_^2^, and λ_3_^2^ and assume without loss of generality λ_1_^2^ ≥ λ_2_^2^ ≥ λ_3_^2^

### Radius of Gyration

The radius of gyration (*R*_g_) can be calculated as

12 $$R_{g}=\sqrt{\frac{1}{N^{2}}<\sum_{i,j=1}^{N}|r_{i}-r_{\mathrm{com}}{|}^{2}>}$$

with *r*_com_ being the center of mass of the NP and N the number of beads in an NP and ⟨·⟩ indicating an ensemble average. This is equivalent to summing up the eigenvalues of the gyration tensor

13 $$R_{g}=\sqrt{\lambda_{1}^{2}+\lambda_{2}^{2}+\lambda_{3}^{2}}$$

### Relative Shape Anisotropy

The relative shape anisotropy κ^2^ of the NPs is computed as

14 $$\kappa^{2}=\frac{3}{2}\frac{\lambda_{1}^{4}+\lambda_{2}^{4}+\lambda_{3}^{4}}{{\left(\lambda_{1}^{2}+\lambda_{2}^{2}+\lambda_{3}^{2}\right)}^{2}}-\frac{1}{2}$$

which ranges from 0 to 1, where 0 is only reached for a perfect spherical symmetry and 1 only in the case of all points lying on a straight line.

### Hydrodynamic Radius

The hydrodynamic radius *R*_h_ of an NP, defined as the radius of a Stokes sphere with the same diffusion coefficient as the NP, can be calculated with the approximate equation^55^

15 $$\frac{1}{R_{h}}=\frac{1}{N\left(N-1\right)}\sum_{i=0}^{N}\sum_{i\neq j}\frac{1}{\left|r_{i}-r_{j}\right|}$$

The sum over *N* items is extended to the beads of the NP and the ions within 3.5σ of any NP bead.

### Charge of Nanoparticles

The total charge of the NPs is simply the sum of all the charges of the NP as well as the charge of all the ions within 3.5σ of any constituent of the NP. The contributions from each component can also be shown separately.

### ζ-Potential

The ζ-potential ζ of an NP is the potential of a test charge at the shear plane, where water and ions no longer travel with the NP but move independently. For the calculation of the ζ-potential, we assume spherical symmetry for our NPs, which is a reasonable assumption for αN/P > 0.9 due to the low shape anisotropy (see Figure 3c). With this assumption, the shear plane should be at *R*_h_ as it is the hydrodynamic radius of the NP including the contact ions moving along with it. The ζ-potential can then be calculated by integrating the electric field along the path of a test charge moved from ∞ to *R*_h_ ^56^

16 $$\zeta =-\int_{\infty }^{R_{h}}\mathrm{E⃗}\cdot d\mathrm{s⃗}=-\int_{\infty }^{R_{h}}\frac{Q\left(r\right)}{4\pi \epsilon_{0}\epsilon_{r}r^{2}}dr$$

with *Q*(*r*) being the charge inside a sphere of radius *r* from the NPs center of mass. We cut the integration at *r* = 50σ, where $\frac{Q\left(r\right)}{r^{2}}\approx 0$

### Figures

Figures were created using Matplotlib^57^ and Gnuplot,^58^ with the “tableau-colorblind10” and “cividis” color scheme specifically chosen to ensure accessibility for all audiences. The python library MDAnalysis^59,60^ was used for most of the analysis of trajectories. VMD^61^ was used for visualization.

## Supplementary Material



## Data Availability

Coordinates and input files of the molecular dynamics simulations, trajectories, and the analysis scripts have been made available on ZENODO (doi:10.5281/zenodo.15364402).

## Notes

Free energy profiles as a function of *n*_NPs_^*^ for systems with different numbers of RNA chains and different PEI length; corresponding parabolic fits; *R*_g_, *R*_h_, and ζ-potential as a function of αN/P ratio; histograms of the number of observed nanoparticles for different αN/P ratios; analysis of the overcharge of nanoparticles; and effective Debye length as a function of αN/P ratio (PDF)

## ∥

NeoVac Ltd., Unit 1 and 2 West, 127 Olympic Avenue, Milton Park OX14 4SA, United Kingdom
