# Supplementary material for: Simulation Insights into the Assembly of Polyplexes for RNA Delivery
Source: Biomacromolecules. 2025 Nov 19;26(12):8465–74. doi: 10.1021/acs.biomac.5c01219 (PMC12690597; doi:10.1021/acs.biomac.5c01219)
Supplement: Supplementary file 1 [file bm5c01219_si_001.pdf]

Supplementary Information for "Simulation Insights into the Assembly of Polyplexes for RNA Delivery"

Jonas Hans Lehnen,[†] Jorge Moreno Herrero,[‡] Heinrich Haas,[‡,¶] Friederike Schmid,[†] and Giovanni Settanni[*,§,†]

†*Department of Physics, Johannes-Gutenberg University Mainz, Germany*

‡*BioNTech SE*

¶*present address, NeoVac Ltd.*

§*Faculty of Physics and Astronomy, Ruhr University Bochum, Germany*

E-mail: giovanni.settanni@rub.de

Phone: +49 (0)234 32 23751. Fax: +49 (0)234 32 14448

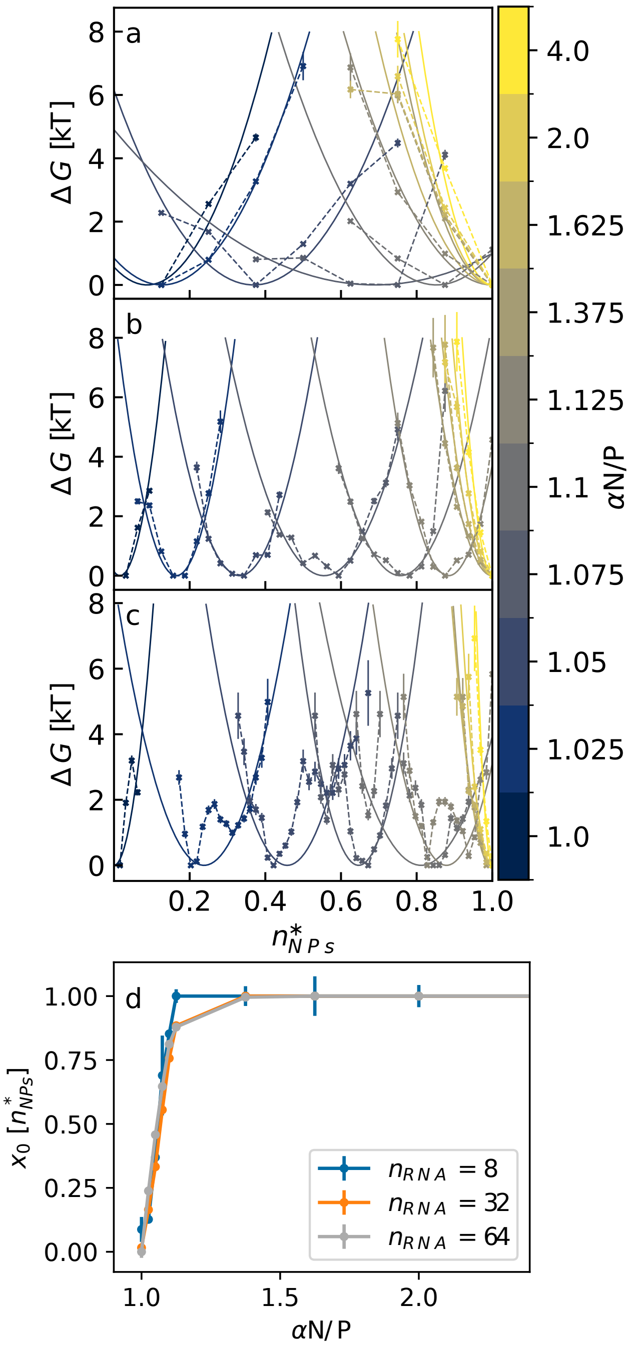

Figure S1: Free energy profiles as a function of n^*_{NPs} for simulations including (a) 8, (b) 32, (c) 64 RNA chains in the box. The overall RNA density is the same across the three panels (the box size changes accordingly). The dashed lines show simulation data while the solid lines represent the fit of harmonic functions. The color scale from blue to yellow indicates increasing αN/P ratios starting from αN/P=1 with blue (see color bar). (d) Position of the minimum of the harmonic approximation of the free energy as a function of αN/P ratio for the simulations including 8 (blue), 32 (orange) and 64 (grey) RNA chains.

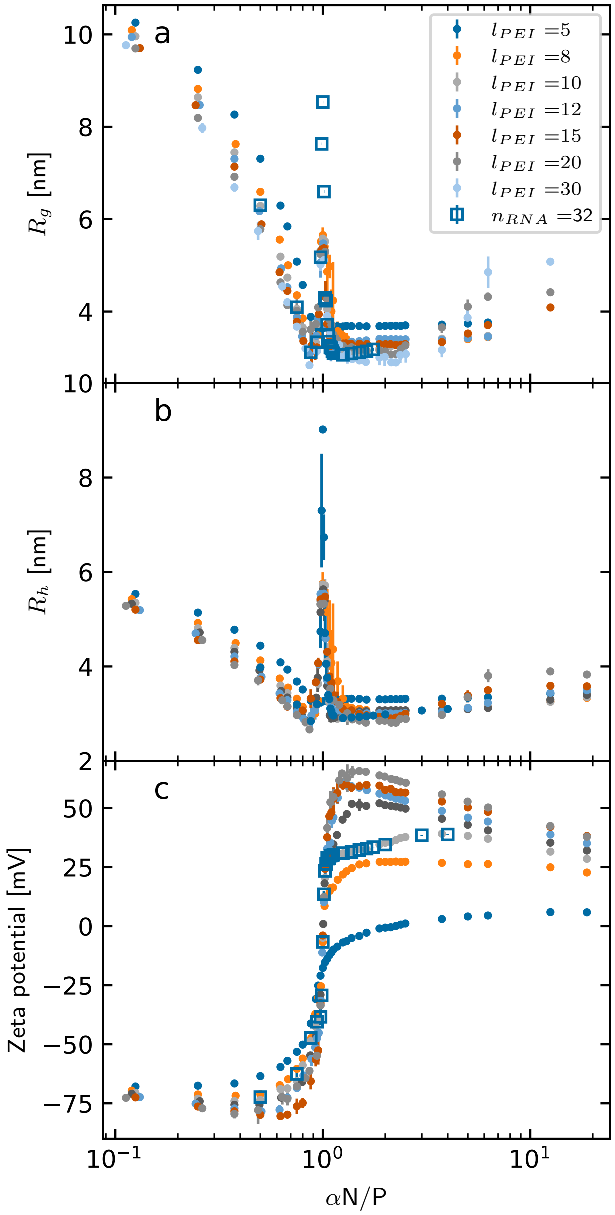

Figure S2: Radius of gyration R_g (a), Hydrodynamic radius R_h (b) and Zeta potential (c) of the simulated NPs. The color of the points reports the length of the PEI chains as indicated. Data from simulations including 8 RNA chains of 100 beads each, with the exception of the squares which report data from simulations including 32 RNA chains and $l_{PEI} = 10$.

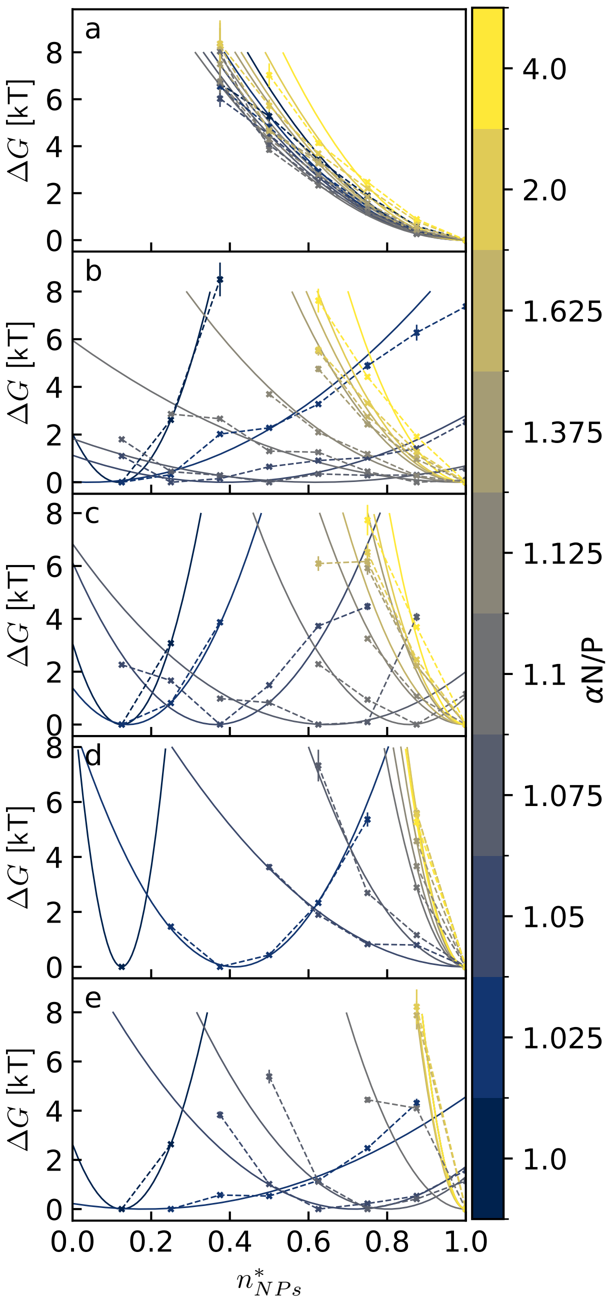

Figure S3: Free energy profiles as a function of n^*_{NPs} for different PEI lengths including (a) $l_{PEI} = 5$, (b) $l_{PEI} = 8$, (c) $l_{PEI} = 10$, (d) $l_{PEI} = 12$, (e) $l_{PEI} = 15$. The dashed lines show simulation data while the solid lines represent the parabolic fit. The color scale from blue to yellow indicates increasing αN/P ratios starting from αN/P=1 with blue (see color bar).

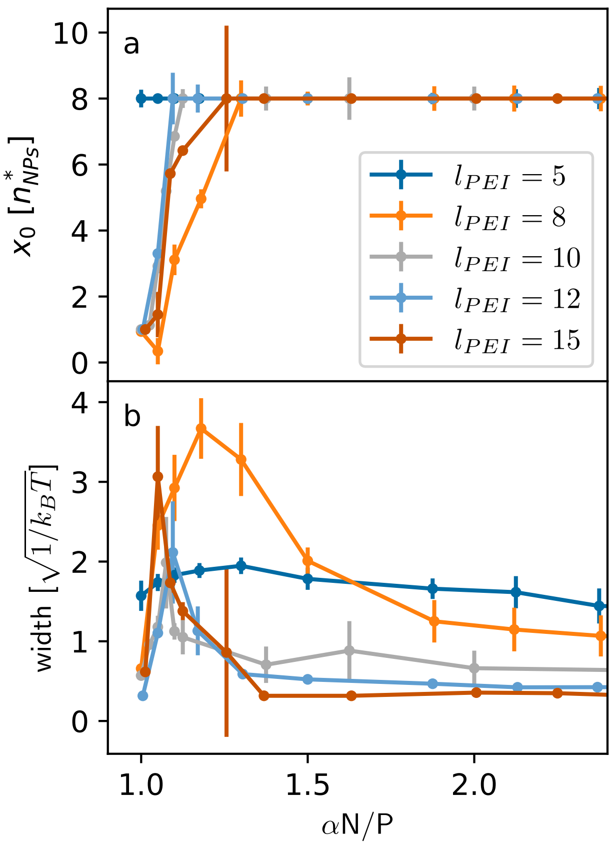

Figure S4: Position of the minimum (a) and width (b) of the parabolic fits of the free energy profiles in Fig. S3 as a function of the αN/P ratio. Data from different l_{PEI} are reported in different colors. The transition from the aggregated state at n^*_{NPs}=0 to the disaggregated state at n^*_{NPs}=1 occurs over a similar range of αN/P ratios at the various PEI length above 10. For the shorter length of 8 the transition range increase and shifts towards larger αN/P ratios and for even shorter length of 5 the fully aggregated states is not observed.

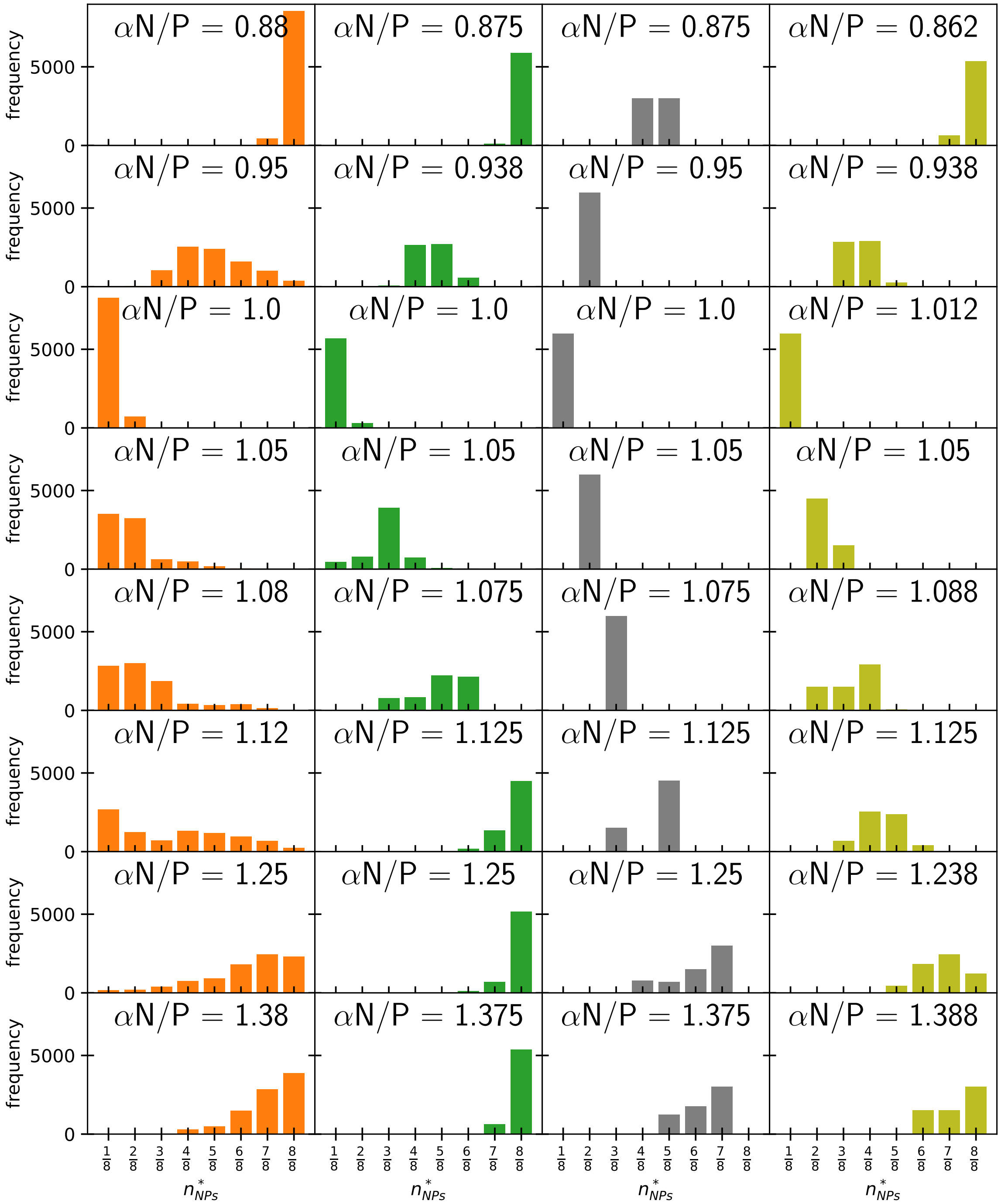

Figure S5: Histograms of the number of observed nanoparticles from simulations made at αN/P ratios close to the midpoint of the aggregation-dissociation transition. Data from simulations where the PEI chain length l_{PEI}=8, 10, 20 and 30 are shown in orange, green, gray and yellow, respectively.

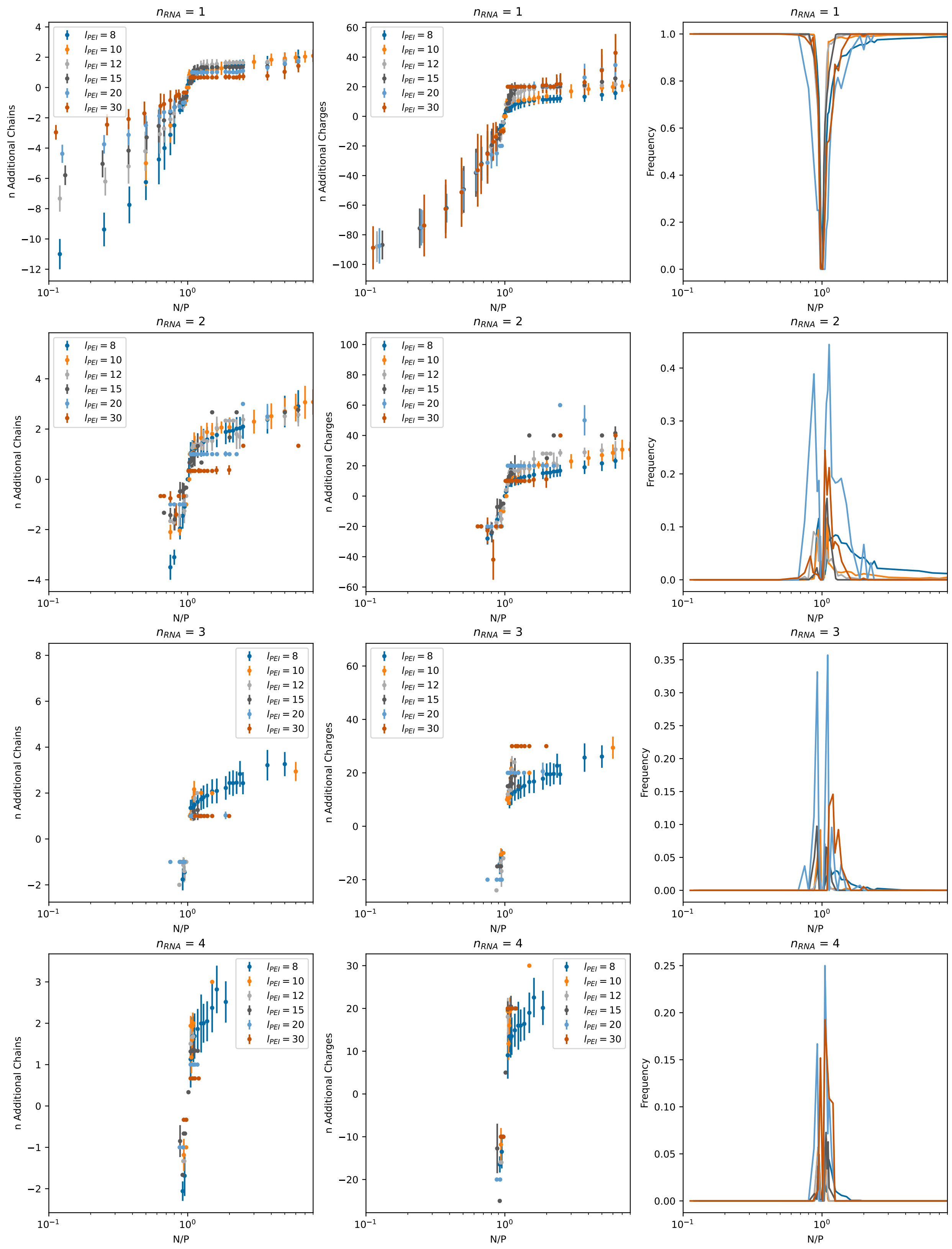

Figure S6: Number of PEI chains (left column) and excess charges (middle) bound to NPs made of a given number of RNA chains and frequency of observation of NPs of that size (right).

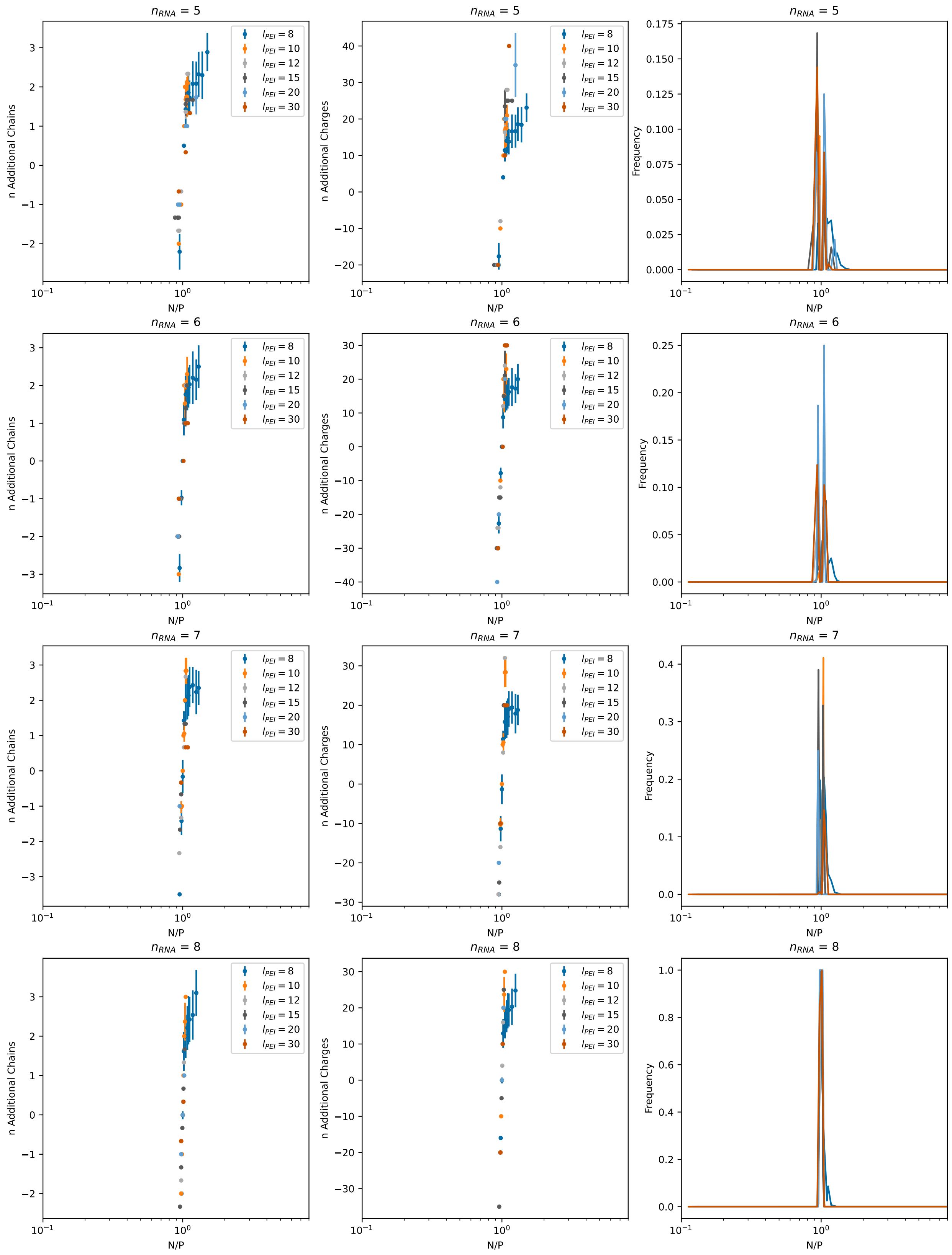

Figure S7: Continuation of Fig. S6.

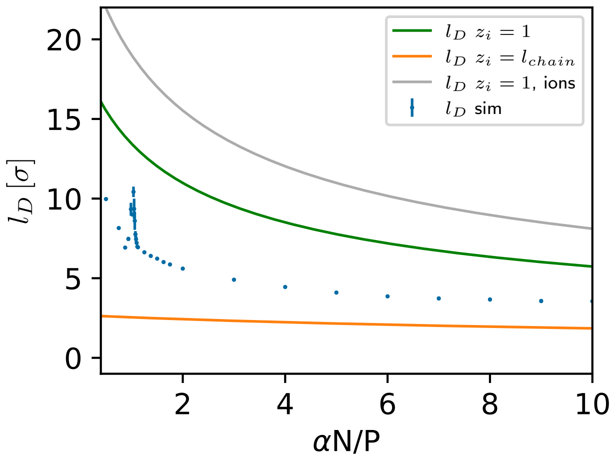

Figure S8: The Debye length in the simulation (blue line) is gathered by fitting the long range decay of the electric potential with $\Psi(r) = \Psi_0 e^{-r/l_D}$. The peak of the Debye length at αN/P≈ 1 is likely caused by the fact that most or all PEI are neutralizing each other in aggregated NPs, reducing the amount of free charges in the simulation. This shows that RNA and PEI chains contribute significantly to the Debye screening length in the simulation. The theoretically estimated Debye length calculated by $l_D = [4\pi l_B \sum_i z_i \rho_i]^{-1/2}$, where l_B is the Bjerrum length, z_i the valence of an ion type and ρ_i the bulk concentration of the type is reported for comparison. The position of the simulation data between the theoretical estimations where every charge is treated like an independent ion (green) and where chains are treated as multivalent ions (orange) shows that the chains in the simulations contribute less to the screening than equivalent multivalent ions, probably due to the more diffuse charge density distribution.
